# Microbial Fermentation Affects the Structure–Activity Relationship of Bioactive Compounds in Ginseng and Its Applications in Fermentation Products: A Review

**DOI:** 10.3390/foods14142473

**Published:** 2025-07-15

**Authors:** Juan Bai, Zixian Zhu, Wei Luo, Miran Jang, Beibei Pan, Ying Zhu, Jiayan Zhang, Yansheng Zhao, Xiang Xiao

**Affiliations:** 1 School of Food and Biological Engineering, Jiangsu University, Zhenjiang 212013, China; 1000005134@ujs.edu.cn (J.B.); 13629445249@163.com (Z.Z.); a32315987371524@outlook.com (W.L.); 17803780879@163.com (B.P.); 1000004369@ujs.edu.cn (Y.Z.); jiayanzhang1988@163.com (J.Z.); zhaoys@ujs.edu.cn (Y.Z.); 2 Department of Institute of Digital Anti-Aging Healthcare, Inje University, Gimhae 50834, Republic of Korea; mrjang@inje.ac.kr

**Keywords:** biotransformation, fermentation, fermented products, ginseng, structure–activity relationship

## Abstract

Microbial fermentation technology has emerged as a pivotal approach for enhancing ginseng efficacy through the transformation of active ingredient molecular structures. This paper reviews the impact of microbial fermentation on the structure–activity relationship of ginseng bioactive compounds and advances in its application. Bibliometric analysis indicates that *Panax* species (*Panax ginseng*, *Panax notoginseng*) are primarily fermented using lactic acid bacteria and *Aspergillus* spp., with research predominantly focused on conversion efficiency to rare ginsenosides (Compound K, Rg3, and Rh2). Specifically, this review details the biotransformation pathways of these rare ginsenosides and the resultant bioactivity enhancements. Additionally, it summarizes the effects of other microorganisms, such as fungal fruiting bodies, on additional ginseng constituents like polysaccharides and polyphenols. Microbial fermentation has been successfully implemented in functional products, including ginseng vinegar, wine, and fermented milk. This review subsequently examines these applications, emphasizing fermentation’s potential to enhance product functionality. However, challenges remain in strain screening, process standardization, and analysis of multi-component synergistic mechanisms. In summary, this review synthesizes recent advancements in understanding the mechanisms of microbial fermentation on ginseng and its translational applications in functional foods and pharmaceuticals.

## 1. Introduction

*Panax ginseng*, a traditional medicinal herb widely utilized in East Asia [1,2], exhibits pharmacological effects, including immune enhancement, anti-fatigue, and anti-aging properties via telomerase activation or ROS reduction [3,4,5]. Its bioactive components, such as ginsenosides, polysaccharides, and polyphenols, are key active substances [6]. However, natural prototype compounds like ginsenosides Rb1 and Rg1 face absorption challenges [7,8,9] due to their complex glycosylation structures and high polarity, which result in low intestinal absorption rates and significant disparity between theoretical activity and practical efficacy. Modern biotechnology plays a pivotal role in overcoming these limitations and advancing ginseng’s therapeutic applications.

Microbial fermentation technology facilitates the transformation of traditional medicinal materials into modern functional products [10,11], offering significant advantages in ginseng fermentation. Common strains include lactic acid bacteria, *Aspergillus* spp., yeasts, *Bacillus* spp., and macrofungi [12], each exhibiting distinctive mechanism of action. Lactic acid bacteria enhance flavor through organic acid production while facilitating ginsenoside transformation [13,14]. Bidirectional solid-state fermentation by large fungi (such as *Ganoderma lucidum* and *Cordyceps sinensis*) and ginseng synergistically enhances levels of active components, promoting saponin conversion while increasing levels of fungal polysaccharides (such as *Ganoderma lucidum* polysaccharides) [15,16,17] and specialized metabolites (such as cordycepin) [18,19,20]. *Aspergillus* spp. secrete β-glucosidase, pectinase, and cellulase, which efficiently decompose the plant cell walls [21,22], significantly improving saponin dissolution and transformation efficiency [23]. Although yeasts and *Bacillus* spp. are less frequently employed, they demonstrate unique capabilities for enriching active components, such as rare saponins and total flavonoids [24,25].

The enzyme system generated through microbial fermentation and metabolism enables targeted modification of ginseng’s active components [26,27,28,29,30], converting them into rare derivatives with enhanced bioavailability and potentiated pharmacological activity [31,32]. For example, β-glucosidase and α-L-rhamnosidase specifically catalyze the deglycosylation of ginsenosides to yield low-glycosylated rare saponins [29,33], significantly augmenting their anti-tumor, immunomodulatory, and neuroprotective functions [34,35]. Furthermore, fermentation reshapes the structure–activity relationship of other bioactive components. This process increases uronic acid and total flavonoid content [36] while enhancing the immunomodulatory efficacy of ginseng polysaccharides and the antioxidant activity of polyphenols [37,38].

Concurrently, growing demand for natural and health-promoting products [39,40] is stimulating the functional diversification of ginseng fermentation products. The product range has expanded beyond traditional forms, such as powders and oral liquids, to novel fermented applications, including dairy products, wines, and vinegars [12,41,42]. These products not only retain the natural properties of ginseng but also acquire new functional properties through microbial metabolism, including enhanced bioactive compound bioavailability and improved flavor profiles [43]. However, current regulatory frameworks governing fermented botanicals, particularly the FDA’s New Dietary Ingredient requirements (21 CFR § 190.6) and the EMA’s reflection paper on fermentation in herbal products (EMA/HMPC/250629/2012), maintain a cautious stance toward novel fermentation methodologies. This necessitates a strategic balance between regulatory compliance and technological innovation.

This article explores the theme of “microbial fermentation affects the structure–activity relationship of bioactive compounds in ginseng and its applications in fermentation products” by providing a systematic review of the latest research progress in this field. Through analyzing the structural modifications and functional enhancements in components such as ginsenosides, polysaccharides, and polyphenols achieved via microbial fermentation, we explored their potential for developing functional products. Using bibliometric analysis and case studies, the paper clarifies the fermentation characteristics by strains of lactic acid bacteria, *Aspergillus* spp., and macrofungi, along with their impact on active components. Current research limitations regarding strain adaptability, metabolic pathway analysis, and standardized processes are highlighted, while future research directions integrating artificial intelligence, bioinformatics, and defined fermentation technologies are proposed. It also proposes future research directions that integrate artificial intelligence (e.g., machine learning for strain selection), bioinformatics, and reference fermentation technology [44,45]. This work provides a theoretical foundation for basic research and industrial applications of microbially fermented ginseng. It also drives the modernization of traditional medicinal materials and advances the big health industry in alignment with SDG 3 (Good Health and Well-being) and SDG 9 (Industry, Innovation, and Infrastructure) through technological innovation and green production models.

## 2. Bibliometric Analysis of Ginseng Fermentation

Bibliometric analysis aggregates and interprets data from peer-reviewed publications to provide an objective framework for delineating research hotspots and developmental trajectories within specialized scientific domains [46,47]. This study employs bibliometric methodologies to conduct a systematic quantitative evaluation of the ginseng fermentation literature, thereby identifying core research domains—including but not limited to ginseng cultivars, microbial consortia, and bioactive phytoconstituents—while mapping the field’s current intellectual architecture and innovation frontiers.

**Figure 1:**
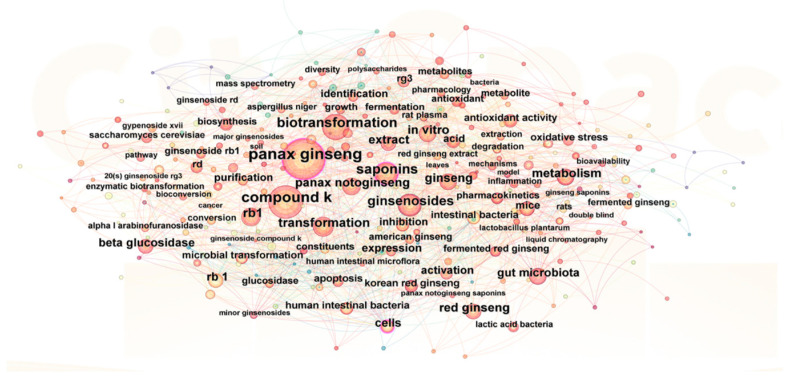
Co-occurrence network of keywords in ginseng fermentation researchers. Node size represents keyword frequency; the thickness of the lines connecting the nodes indicates the degree of association between the keywords.

The Web of Science Core Collection database was systematically searched for publications pertaining to the topics “ginseng fermented,” “*Panax quinquefolius* fermentation,” “*Pnax ginseng* fermentation,” and related variants. The search covered the period from 1 January 2000 to 1 April 2025. Conference proceedings, newspaper articles, and publications deemed to have low relevance to the research topic were excluded from consideration. Duplicate records were also eliminated, resulting in a final dataset comprising 770 pertinent publications. CiteSpace (v6.3.R1) was utilized for visualization and analysis purposes. The software parameters were configured as follows: a time slicing interval of three years; node type set to Keyword; top N limited to 50; and both Pathfinder and Pruning algorithms applied for network pruning within the merged network. Cluster analysis was conducted using the Log-Likelihood Ratio (LLR) algorithm. Keywords with a frequency of ≥13 were selected to generate a keyword co-occurrence map (Figure 1). In this map, nodes of varying colors—representing distinct clusters—are interconnected by lines. Each node corresponds to a keyword, with node size reflecting its frequency in the literature [48].

Based on the CiteSpace (v6.3.R1) analysis, we performed statistical profiling of ginseng varieties, microorganisms, and active components. As depicted in Figure 1 and Table 1, *Panax ginseng*, *Panax notoginseng*, and American ginseng (*Panax quinquefolius*) constitute the three most extensively studied *Panax* species in fermentation research. Regarding microorganisms, lactic acid bacteria (LAB), *Aspergillus* species, and *Ganoderma lucidum* predominate, with LAB applications constituting nearly 50% of reported cases. Research primarily focuses on key ginseng constituents, particularly ginsenosides, where Compound K and Rb1 emerge as major investigative targets. Beyond ginsenosides, a subset of studies examines additional active compounds, including ginseng polysaccharides and polyphenols.

## 3. Utilization of Different Microorganisms in Ginseng Fermentation

The microorganisms employed in the fermentation of ginseng encompass lactic acid bacteria, *Aspergillus* spp., yeasts, and macrofungi, as shown in Table 2. Among these microorganisms, lactic acid bacteria (comprising *Lactiplantibacillus plantarum*, *Limosilactobacillus fermentum*, *Lactobacillus helveticus*, and *Lacticaseibacillus paracasei*, amongst others) demonstrate the most extensive utilization. *Aspergillus* spp. and macrofungi represent the next most prevalent taxa, whereas yeasts and *Bacillus* spp. exhibit comparatively limited application in ginseng fermentation, which may be attributed to the safety concerns associated with probiotics derived from *Bacillus* spp., as well as the influence of metabolic by-products produced by yeasts, such as ethanol, on the fermentation products [49,50]. Strain selection must align with specific fermentation objectives and methodologies. The current focus of research in the field of microbial fermentation is primarily directed toward enhancing the content of ginseng active ingredients, such as rare ginsenosides and total polyphenols, thereby enhancing the pharmacological activity of ginseng both in vivo and in vitro [51,52].

### 3.1. Lactic Acid Bacteria

Owing to their potential probiotic traits, lactic acid bacteria (LAB) serve as essential starters in fermenting dairy products (e.g., fermented milk and cheese) and vegetables (e.g., kimchi) [40,53,54,55]. Their recent application in ginseng fermentation has garnered increasing attention. LAB fermentation significantly elevates both the content and bioavailability of the active compounds found in ginseng [56]. Ginsenosides serve as the principal bioactive constituents of ginseng, yet the major forms naturally present in plants exhibit limited bioavailability due to poor absorption in humans [7,9]. During fermentation, β-glucosidase secreted by lactic acid bacteria can hydrolyze major ginsenosides, such as Rb1 and Rg1, into rare ginsenosides, like Rg3, F2, and Rh2 [38,57,58], which demonstrate superior absorption. The increase in the concentrations of rare ginsenosides significantly enhances the physiological activities of ginseng, including anti-tumor effects, antioxidant properties, neuroprotection, and immune regulation [3,4,6]. This process further improves the bioavailability of ginseng [59,60,61].

Furthermore, lactic acid bacteria generate substantial quantities of lactic acid and other organic acids during fermentation [62,63,64]. These acidic compounds can mitigate the bitterness of ginseng and enhance the palatability of ginseng-based fermented products, as confirmed by sensory evaluation from consumer panels [12,13,65,66,67], thereby offering innovative perspectives for the development of functional ginseng products.

In conclusion, lactic acid bacterial fermentation not only enhances the nutritional value of ginseng but also opens up new avenues for its use in functional foods and pharmaceuticals. However, screening and optimizing lactic acid bacteria strains, standardizing fermentation processes, and precisely regulating metabolic products (such as detecting saponin isomers and monitoring metabolic dynamics) remain key areas for future research.

### 3.2. Aspergillus spp.

As a significant industrial microorganism, *Aspergillus* spp. exhibits unique metabolic advantages and biotransformation potential in ginseng fermentation [23]. Currently, the *Aspergillus* species utilized in ginseng fermentation include *Aspergillus niger*, *Aspergillus tubingensis*, and *Aspergillus oryzae*, with *Aspergillus niger* being the most widely employed [12]. These fungi secrete highly active enzymes, such as pectinase, cellulase, and xylanase [68,69,70,71]. During fermentation, these enzymes decompose the plant cell walls on the surface of ginseng, releasing bioactive compounds like saponins and polysaccharides that are encapsulated within [72,73,74]. Additionally, β-glucosidase secreted by *Aspergillus* spp. can convert primary ginsenosides into rare ginsenosides, thereby significantly enhancing the anti-tumor and immune-regulating properties of fermented ginseng [23,75,76,77]. For example, Ramadhania et al. found that fermentation of black ginseng by *Aspergillus niger* KHNT-1 resulted in significantly enhanced anti-melanogenesis, anti-wrinkle, and antioxidant activities [78].

Although the *Aspergillus* spp. enzyme system is rich [68,69] and shows unique advantages in the fermentation of ginseng, it is difficult to separate the mycelium produced after fermentation from the substrate [79], and the process for extracting the target product is rather complex. Furthermore, the spores produced by *Aspergillus* spp. are highly heat-resistant and difficult to inactivate [80], potentially remaining in the final product and causing secondary fermentation or deterioration. In light of the aforementioned limitations, it may be beneficial to explore solid-state fermentation technology and inert carriers in future endeavors [81,82]. These approaches could enhance mycelium separation and broaden the application scope of *Aspergillus* spp. in ginseng fermentation [83].

### 3.3. Macrofungal

Bidirectional solid fermentation of macrofungi, such as *Ganoderma lucidum*, *Cordyceps militaris*, and *Schizophyllum* spp., in combination with ginseng has emerged as a significant research area in recent years [12,84]. Bidirectional fermentation alters the profile of ginsenosides and elevates the levels of active components within the fungi, creating a synergistic effect between the fungi and ginseng constituents. For example, co-fermentation of *Ganoderma lucidum* with ginseng promotes the enrichment of various secondary metabolites, such as polysaccharides and triterpenoids, in *Ganoderma lucidum* [85]. These fungal metabolites subsequently synergize with the active components of ginseng to augment physiological functions, including antioxidant, anti-inflammatory, and immune-regulatory properties [85,86,87]. Similarly, co-fermentation of Cordyceps militaris with ginseng not only increases the content of rare ginsenosides but also promotes the synthesis of cordycepin, resulting in multiple combined physiological effects [18,88,89].

This bidirectional fermentation system can significantly increase the yield of active ingredients or generate new metabolites through the synergistic effect of fungi and medicinal plants, providing new ideas for the development of functional foods and drugs, but problems such as slow mycelial growth rate and long fermentation cycles still need to be solved through process optimization. In future endeavors, metabolic engineering techniques could be employed to modify fungal strains to improve utilization efficiency and product specificity within this fermentation system [90].

**Table 2:** Microbial fermentation of ginseng substrates and its products.

Microorganisms	Substrates	Products	Outputs	References
*Aspergillus tubingensis* KCTC 14,166	American ginseng extract	C-K	8.06 g/L	[23]
*Rhizopus oligosporus*	Wild Ginseng	Total saponins	2299 mg/kg	[91]
		Total phenolic	5.65 ± 0.72 mM GAE/g	
		L-Carnitine	630 mg/kg	
*Lacticaseibacillus paracasei* B16NY2107 and B04WI2501	*Panax ginseng*	Rg3	92.981 ± 3.188 mg/L	[92]
*Saccharomyces cerevisiae* F6	Ginsenoside extract	Rh4	2.65 mg/g	[93]
		Rg5	2.56 mg/g	
*Cordyceps militaris* KCCM 60304	Red ginseng	Rb3	9.16%	[18]
		Rd	513.93%	
		Rg2	63.12%	
		Rg3 (20S)	101.17%	
		Rg3 (20R)	112.53%	
		cordycepin	34.8 mg/kg	
*Bacillus subtilis* CCTCC M 2,020,002 and *Trichoderma reese* CICC 2626	Ginseng powder	Total saponins	21.79 mg/g	[94]
*Lactiplantibacillus plantarum* B1	Ginseng extract	C-K	0.7706 mg/kg	[38]
		Rk1	0.7348 mg/kg	
		Rh4	3.3924 mg/kg	
		Rg5	1.3648 mg/kg	
*Saccharomyces cerevisiae* GIW-1	*Panax ginseng*	Uronic acid	-	[36]
		Acidic polysaccharide	-	
*Lactiplantibacillus plantarum* KCCM 11613P	*Panax ginseng* Meyer	Rd	55.74 ppm	[52]
		Total phenolic	37.67 ± 0.37 mg GAE/g	
*Aspergillus awamori*	Black ginseng	Acidic polysaccharide	74.2%	[77]
		Rg3, Rg5, and Rk1	4.13 mg/g	
*Bacillus licheniformis* IDCK 30 and *Bacillus subtilis* IDCK 40	Mountain-cultivated ginseng	Rg3	166.90 μg/g	[95]
		C-K	231.33 μg/g	
*Aspergillus tubingensis* KCTC 14166	American ginseng extract	C-K	17.1 mg/L/h	[76]
*Leuconostoc mesenteroides* KCCM 12010P	Hydroponic ginseng	Total phenolic	107.19%	[96]
		Total flavonoid	645.59%	
*Lactiplantibacillus plantarum* MB11	ginsenoside extract	Rh2	62.37 mg/g	[97]
*Cordyceps militaris* KCCM 60304	Korean red ginseng	Rd	2.23 ± 0.28 mg/g	[89]
		Rg3	3.50 ± 0.29 mg/g	
*Monascus pilosus* KMU103	Red ginseng	Rh1, Rh2, Rg3	838.7 mg/kg	[98]
		Monacolin K	3089 mg/kg	
*Chaetomium* sp. F24-W and *Aspergillus niger*	*Panax notoginseng*	Rg3	108.95 mg/L	[99]

“-” indicates absence.

## 4. Biotransformation and Structure–Activity Relationship of Bioactive Compounds in Fermented Ginseng

Biotransformation refers to the process of converting substrates into higher-value products through the utilization of biological catalysts, including enzymes, microorganisms, and animal or plant cells [100,101,102]. The predominant focus of microbial fermentation in ginseng processing is the biotransformation of ginsenosides. Through the process of microbial fermentation, the main ginsenoside in ginseng can be transformed into rare ginsenosides, which possess higher physiological activity. Furthermore, the impact of microbial fermentation on the structure–activity relationship of other bioactive substances, including polysaccharides and polyphenols, remains underexplored.

### 4.1. Ginsenoside

Ginsenosides are triterpenoid compounds constituting the primary bioactive constituents of *Panax* plants [103]. Their core structures are characterized as dammarane or oleanane types [7,104]. Glycosylation modifications yield diverse derivatives, which are categorized into two classes based on sugar moiety composition: prototype ginsenosides (e.g., Rb1 and Rg1) and rare ginsenosides (e.g., Rg3, Rh2, and F2) [56,105]. Studies indicate that both the number and positional configuration of glycosides attached at different sites on the aglycone skeleton critically determine biological activity [56,103]. Specifically, reduced glycosylation (as in monoglycosides) and terminal glycoside hydrolysis products (rare ginsenosides) consistently demonstrate enhanced pharmacological effects and superior bioavailability [8,9]. For instance, ginsenoside Rh2 (a monosaccharide) demonstrates markedly greater anti-tumor activity than Rg3 (a disaccharide), primarily attributable to Rh2’s superior cell membrane permeability and ability to disrupt cancer cell signaling pathways [106,107].

Owing to the limited natural abundance of rare ginsenosides with superior bioactivity (e.g., Rg3 and CK) in *Panax* plants [103,107], microbial fermentation technology has garnered significant research interest for converting major ginsenosides (such as Rb1) into these high-value derivatives [56]. This bioprocess addresses the growing demand for clinical applications by enhancing ginsenoside bioactivity. The transformation mechanism primarily involves microbial secretion of β-glucosidases and β-arabinofuranosidases, which selectively hydrolyze glycosyl groups at C3, C6, and C20 positions on the aglycone core, thereby facilitating rare saponin biosynthesis [108,109].

Damaran-type (DM-type), oleanolic acid-type (OA-type), and oxytetracycline-type (OCT-type) ginsenosides are the three main structural types of ginsenosides at present, among which damaran-type ginsenosides account for the vast majority, and the latter two structural types of ginsenosides are rarely found in nature [103,109]. According to the differences in glycosides and hydroxyl ligands at C3, C6, and C20, the DM-type ginsenosides can be divided into proto-panaxanadiol type (PPD-type) ginsenosides and protopanaxtriol type (PPT-type) ginsenosides [56]. PPD-type ginsenosides are formed by the combination of β-OH and a glycogroup at C3 or C20 of PPD, mainly including Ra1, Ra2, Ra3, Rb1, Rb2, Rb3, Rc, Rd, Rg3, and Rh2. PPT-type ginsenosides are formed by the combination of α-OH at C6 of PPT or β-OH at C20 with glycogroups, mainly including Re, Rf, Rg1, Rg2, Rh1, F1, R1, and R2 [56,110].

(1) PPD type

**Figure 2:**
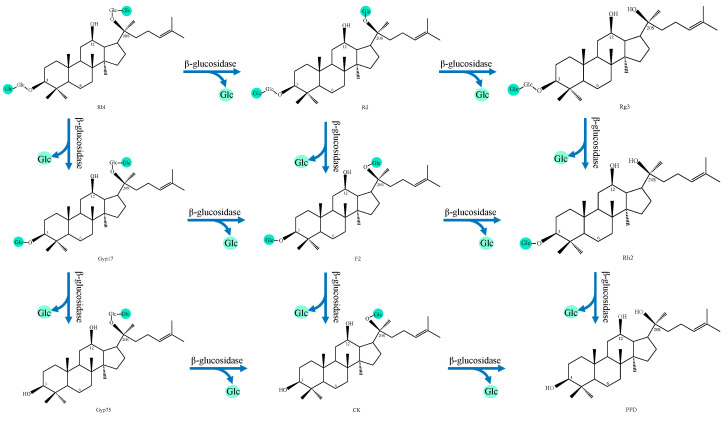
The biotransformation pathway of Rb1.

Rb1 is the most prevalent compound in the biotransformation of PPD-type ginsenosides, primarily due to its higher abundance in ginseng extracts [30]. Undergoing deglycosylation by β-glucosidase, Rb1 can be converted into various secondary ginsenosides through distinct hydrolysis pathways, as shown in Table 3 and Figure 2, ultimately yielding the aglycone protopanaxadiol (PPD) upon complete conversion. The types and activities of β-glucosidases produced during metabolism vary depending on different microbial species [111]. Based on linkage location, residue structure, and enzyme activity, glucosidases can be classified into four categories [112]: Type I glucosidase is capable of simultaneously hydrolyzing the glycosyl groups linked at the C3 and C20 positions of PPD ginsenosides; Type II glucosidase specifically targets the glycosyl group at the C20 position of either PPD or PPT ginsenosides; Type III glucosidase acts on glucose located at the lateral C3 junction of PPD ginsenoside; and lastly, Type IV glucosidase hydrolyzes the glycogroup linked to the C6 in PPT ginsenoside [56,112].

Most current studies focus on microbial conversion of Rb1 to the rare ginsenosides Rg3, Rh2, or C-K. For example, crude β-glucosidase URN103L from *Lactobacillus buchneri* can convert Rb1 to Rg3 [111], and *Lactiplantibacillus plantarum* OG-05, *Leuconostoc citreum LH1*, and *Lactobacillus brachys* THK-D57 can convert Rb1 to C-K via pathway 4 [113,114,115]. As indicated in Table 3, ginsenoside C-K can also be produced from Rb1 via pathway 2. *Leuconostoc mesenteroides* DC102 and *Leuconostoc lactis* DC201 co-cultures and *Companilactobacillus paralimentarius* LH4 can degrade Rb1 to ginsenoside C-K through intermediates Gyp17 and F2 [116,117]. In addition, some studies have reported that Rb1 cannot be completely converted to C-K in the process of microbial fermentation, resulting instead in the accumulation of intermediates ginsenosides, such as Rd or Gyp17. *Bifidobacterium dentium* and *Lacticaseibacillus rhamnosus* GG were only able to convert Rd or Gyp17 during the fermentation of Rb1 [118,119], suggesting that the β-glucosidase activity in these bacteria is limited to the hydrolysis of the β-D-glucoside linkage at either C3 or C20, rather than both positions.

(2) PPT type

**Figure 3:**
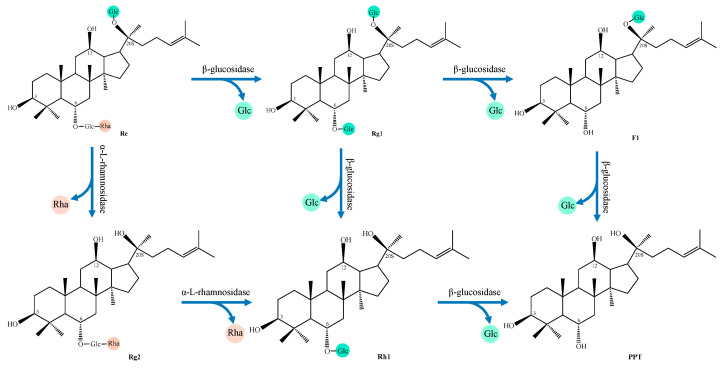
The biotransformation pathway of Re.

The content of PPT-type ginsenosides in natural *Panax* plants is significantly lower than that of PPD-type ginsenosides [56], so there are relatively few research reports on the fermentation transformation of PPT-type ginsenosides. Ginsenoside Re and Rg1 are the predominant initial substrates for PPT-type ginsenoside biotransformation [120]. Notably, Rg1 can be derived from Re through hydrolysis of the terminal α-L-rhamnose moiety at the C6 position of ginsenoside Re by α-L-rhamnosidase. [107]. Figure 3 and Table 4 provide a comprehensive summary of the various transformation pathways of ginsenoside Re. Most reports show that ginsenosides Re and Rg1 are mainly converted to the rare ginsenoside Rh1 via glycosidase hydrolysis. For example, the conversion rate of ginsenoside Rh1 produced by ginsenoside Rg1 after fermentation with *Cordyceps sinensis* from different sources can reach 54.9% and 82.5% [121,122]. The co-culture of *Leuconostoc mesenteroides* YLB8, *Lactobacillus helveticus* KII13, and *Pediococcus pentosaceus* KID7 can convert ginsenoside Re to Rh1 via pathway 1 [123,124].

In conclusion, microbial fermentation can significantly modulate the structure–activity relationship of ginsenosides. Table 5 provides a comprehensive summary of the transformation pathways associated with various types of ginsenosides. Rare ginsenosides generated through microbial fermentation and transformation, such as C-K, Rg3, and Rh1, contain fewer glycosides and are more easily absorbed and utilized by the human body. These ginsenosides demonstrate more significant neuroprotective, anti-inflammatory, antioxidant, and anti-tumor activities [120,125,126]. Subsequently, we can use gene editing and synthetic biology techniques to optimize the key enzymes in the strain that convert ginsenosides. This will enhance the synthesis efficiency of rare ginsenosides and accelerate their industrial-scale production.

### 4.2. Ginseng Polysaccharide

In recent years, alongside the research focus on core active ingredient ginsenosides, the structure–activity relationship of ginseng polysaccharides has gained increasing attention. Ginseng polysaccharides primarily comprise ginseng pectin and ginseng amyloid substances. Ginseng pectin is recognized as the principal active component within ginseng polysaccharides [137]. It predominantly consists of an acidic heteropolysaccharide mixture that includes galactose (Gal), galacturonic acid (GalA), rhamnose (Rha), and arabinoxylan (AX), among others. This mixture encompasses arabinogalactan (AG), rhamnogalacturonic acid (RG), and homogalacturonic acid (HG). The main constituent of ginseng starch is amyloid glucan, which is composed of α-D-(1,4)-glucan, 6-branched α-D-(1,4)-glucan, 3-branched α-D-(1,6)-glucan, and non-side chain α-D-(1,6)-glucan [138]. Currently identified types of ginseng polysaccharides include water-soluble polysaccharide (GR-4), acidic polysaccharides (SA, SB, PA, PB, ginseng-I A; ginseng-II A, GR-5AUL, GR-5AUH, and PG-F2), and alkali-soluble polysaccharides (GRA-3 and GRA-4) [139].

The bioactivity of ginseng polysaccharides is modulated by their compositional profile, structural features, and concentration [140]. For example, Chen et al. [141] demonstrated that ginseng aerial part-derived polysaccharides exhibit significantly higher antioxidant activity than subterranean part-derived counterparts, with neutral polysaccharides > acidic polysaccharides in efficacy. Furthermore, Zhang et al. [142] demonstrated that arabinogalactan (AG) side chains in *Panax ginseng*-derived polysaccharide WGPA-2-RG critically mediate nitric oxide secretion and lymphocyte proliferation.

Microbial fermentation significantly modulates the structure–activity relationship of ginseng polysaccharides. Kim et al. [143] fermented ginseng using *Hericium erinaceum* mycelia, revealing that crude polysaccharides from fermented ginseng (FG-HE-CP) exhibit superior immunomodulatory activities, including enhanced splenocyte proliferation, macrophage activation, and gut immunoregulation, compared to unfermented extracts. Critically, this study demonstrated the essential role of hydroxyl groups in polysaccharide immunogenicity [143]. Furthermore, fermentation of ginseng polysaccharides by *Saccharomyces* GIW-1 enhanced antioxidant activity through improved hydroxyl radical scavenging, superoxide anion elimination, and total antioxidant capacity [36]. These fermented products also alleviated lipopolysaccharide-induced liver inflammation in mice, with Ai et al. attributing this hepatoprotective effect to increased glucuronic acid content [36]. Similarly, You et al. [144] fermented *Panax notoginseng* with *Saccharomyces cerevisiae* CGMCC 17452, significantly increasing the polysaccharide yield. The resulting fermented notoginseng polysaccharide (FPNP) inhibited H_2_O_2_-induced damage to collagen and elastic proteins by activating the TGF-β/Smad signaling pathway, thereby protecting against oxidative stress [144].

Currently, over 80 distinct sugar moieties have been isolated from ginseng polysaccharides [145], many exhibiting significant immunomodulatory [146], antioxidant [147], and anti-tumor properties [148,149]. However, due to the high molecular weight of ginseng polysaccharides, elucidating their complete structures using chemical and spectral techniques remains challenging. Consequently, the structure–activity relationship of ginseng polysaccharides is not yet fully understood; extensive research is required in the future to determine their structural characteristics. Future research should integrate molecular dynamics simulations with metabolomics to establish quantitative correlations between specific sugar chain modifications and their biological activities.

### 4.3. Polyphenols

In addition to the significant presence of saponins and polysaccharides, *Panax* plants also contain various polyphenols, including flavonoids. Although these compounds are present in relatively low concentrations, they play crucial roles in the biological activity of ginseng, contributing notably to its antioxidant [150] and hypoglycemic properties [16].

Polyphenols in plants can also undergo biotransformation following microbial fermentation [151]. For instance, certain flavonoids exist as glycosides, such as quercetin and kaempferol [152]. The β-glucosidase secreted by microorganisms can hydrolyze the glycosidic bond and release aglycones. For instance, quercetin glycoside is converted to quercetin by microbial fermentation, and its 3-OH and 4′-OH free hydroxyl groups significantly enhance the ability to scour free radicals [48]. Studies confirm that aglycone flavonoids exhibit 2–3 times greater antioxidant activity than their glycosylated forms. [153].

Current research reports demonstrate that elevated total phenol and flavonoid levels during ginseng fermentation enhance antioxidant activity through improved free radical scavenging capabilities [95,154,155]. For instance, Liu et al. conducted a fermentation of ginseng extract using *Lactiplantibacillus plantarum*, and their findings revealed that the concentrations of total phenol and total flavone in the ginseng extract increased post-fermentation, resulting in a fermented ginseng extract with markedly stronger antioxidant activity [38].

Current research on ginseng polyphenols remains limited, leaving the specific mechanisms through which fermentation modulates the structure–activity relationships incompletely characterized. Future studies should combine with metabolomics and molecular docking technology to accurately analyze the mechanism of action of key active groups and to explore synthetic biology methods for the directed synthesis of highly active compounds.

## 5. Ginseng Fermented Products

With the advancement of microbial fermentation technology, the application of ginseng in health food has progressively transitioned from basic products, such as ginseng powder, ginseng honey slices, and ginseng oral liquid, to more sophisticated functional fermented products, including ginseng fermented wine, ginseng fermented milk, ginseng vinegar, and ginseng fermented beverages, as shown in Table 6. This evolution contributes to improved utilization of ginseng while supporting increased added value and expanded market potential.

### 5.1. Ginseng Fermented Wine

The current market features diverse fermented ginseng wines, including rice wine, beer, fruit wine, and soju. Ginseng fermented wine not only maximizes the retention of ginseng’s original benefits and is rich in nutrients but also contains a moderate alcohol content. It boasts a smooth taste along with the unique aroma and flavor of ginseng, making it refreshing and enjoyable for a wide range of consumers. Park et al. developed ginseng beer by incorporating red ginseng extract fermented with *S. cerevisiae* Saflager W-34/70 as the fermentation agent [156]. Sensory evaluation results indicated that fermentation at 4 °C for 21 days yielded the highest scores in terms of flavor, taste, and overall acceptability. Pyo et al. [157] fermented *Panax ginseng* sprouts with brewing yeasts to produce ginseng fermented wine (GFW) and analyzed its physicochemical properties. Fermentation increased acidity, total phenol content, and ABTS radical scavenging activity, whereas pH and the reducing sugar content decreased. Notably, ginsenoside Re was the predominant ginsenoside in GFW, followed by Rg1 and Rh1; other ginsenosides were present in trace amounts [157]. Therefore, ginseng fermented wine not only preserves its original flavor but also offers antioxidant properties and other beneficial functions. As health-conscious consumer demand grows, future product development should target specialized segments through optimized fermentation protocols.

### 5.2. Ginseng Fermented Milk

Ginseng is extensively incorporated into dairy products, particularly fortified milk, fermented milk, and cheese, with ginseng fermented milk being the predominant fermented variant. Jung et al. [158] produced fermented milk supplemented with red ginseng extract (0.5%, 1%, 1.5%, and 2%) using *Lactobacillus acidophilus* and *Streptococcus thermophilus* and found that the antioxidant effect of the fermented milk was directly proportional to the concentration of red ginseng extract. Lee et al. [66] developed a variant of ginseng fermented milk using hydroponic ginseng (HG). The results showed that the sensory scores of color, flavor, texture, taste, and overall acceptability of fermented milk with 0.5% HG addition had no significant difference from that of the fermented without HG addition (control), but the fermented milk fortified with HG extract exhibited higher antioxidant activity than the control group [66]. In another study, Cimo et al. [159] produced fermented milk containing American ginseng roots, and the results indicated that ginseng enhanced the survival rate of *Lacticaseibacillus rhamnosus* GR-1, while the starter culture increased the content of ginsenosides Rg1, Re, Rb1, and Rb2 in ginseng [159]. The aforementioned studies underscore that ginseng can be effectively integrated into high-value dairy products while serving as a health supplement endowed with bioactive properties within the food industry.

### 5.3. Ginseng Vinegar

Ginseng vinegar represents another prominent type of ginseng fermentation product. The traditional production process of ginseng vinegar is mostly vinegar soaking, but the introduction of modern fermentation technology has greatly improved its ingredient utilization and functional properties. Baik et al. [160] used rice wine and red ginseng concentrate (RGC) as substrates to produce red ginseng vinegar by adding *Acetobacter aceti*. The sensory evaluation results showed that the flavor, taste, and overall preference of red ginseng vinegar received high scores. In another study, Yeo et al. [161] used hydroponic ginseng to develop hydroponic ginseng vinegar (HGV) containing ginsenoside Rg2. Compared with the control group, the level of ginsenoside Rg2 increased 4.0-fold in acetic acid-fermented HGV, and HGV had significant anti-obesity and anti-hyperlipidemic effects. The health benefits of traditional vinegar are somewhat limited. However, the addition of ginseng vinegar endows it with the attribute of “food and medicine sharing the same origin,” and its antioxidant properties can extend the shelf-life of food while enhancing the nutritional value of dishes.

**Table 6:** Ginseng fermented products.

Product Names	Starter Cultures	Functions	References
Ginseng wine	*Saccharomyces cerevisiae*	Hepatoprotective effect	[162]
Ginseng alcoholic drink	*Saccharomyces cerevisiae*, *Saccharomyces bayanus*	-	[163]
Ginseng wine	*Saccharomyces cerevisiae*, *Saccharomyces carlsbergensis*	-	[164]
Red ginseng wine	*Saccharomyces cerevisiae*	-	[165]
*Panax ginseng* sprout wine	*Saccharomyces cerevisiae*	Antioxidant	[157]
Ginseng beer	*Saccharomyces cerevisiae*	-	[156]
Ginseng makgeolli	*Saccharomyces cerevisiae*	-	[166]
Ginseng rice wine	Kefir grain	-	[167]
Ginseng makgeolli	*Saccharomyces cerevisiae*	-	[168]
Ginseng-cactus wine	*Saccharomyces cerevisiae*	Anti-fatigue	[169]
Ginseng fermented milk	*Lactobacillus acidophilus*, *Streptococcus thermophilus*	Antioxidant	[158]
Ginseng fermented milk	*Lactobacillus acidophilus*, *Bifidobacterium longum* subsp. *longum*, *Streptococcus thermophilus*	Antioxidant	[66]
Ginseng fermented milk	*Lacticaseibacillus rhamnosus* GR-1, *Lactobacillus delbrueckii* subsp. *bulgaricus*, *Lactobacillus delbrueckii*, *Streptococcus thermophilus*	-	[159]
Ginseng fermented milk	*Lactiplantibacillus plantarum* NK181, *Streptococcus thermophilus*	Antioxidant	[170]
Ginseng fermented milk	*Lactiplantibacillus plantarum* SY46, *Levilactobacillus brevis* SY65	Antioxidant	[171]
Ginseng fermented milk	*Lactobacillus delbrueckii* subsp. *bulgaricus*, *Streptococcus thermophilus*, *Bifidobacterium bifidum*	Antioxidant	[172]
Ginseng fermented milk	*Bifidobacterium minimum* KK-1, *Bifidobacterium cholerium* KK-2	-	[173]
Ginseng fermented milk	*Lactobacillus acidophilus* KCTC3150, *Ligilactobacillus salivarius* ssp. CNU27	-	[174]
Ginseng vinegar	*Acetobacter aceti*	-	[160]
Ginseng vinegar	*Acetobacter aceti*	Anti-obesity	[161]
Ginseng persimmon vinegar	*Acetobacter aceti*	Lipid-lowering effect	[175]
Ginseng vinegar	Mix microbial powder	-	[176]
Ginseng vinegar	*Acetobacter pasteurianus* JBA190503	Anti-inflammatory effect	[42]
Ginseng vinegar	*Acetobacter aceti*	Antioxidant	[177]
Ginseng fruit vinegar	*Acetobacter aceti*	-	[178]
Ginseng-prunus mume fruit vinegar	*Acetobacter aceti*	Anti-fatigue effect	[179]
Effervescent tablets of lactobacilli	*Lactobacillus acidophilus* *Lacticaseibacillus rhamnosus* *Lactiplantibacillus plantarum*	-	[180]
Ginseng cheese	*Lactobacillus acidophilus*	-	[181]
Ginseng cheese	Flora Danica (*Lactococcus lactis* subsp. *lactis*, *Lactococcus lactis* subsp. *cremoris*, *Lactococcus lactis* subsp. *lactis* biovar diacetylactis, *Leuconostoc mesenteroides* subsp. *cremoris*)	-	[182]
Ginseng fermented milk	*Ligilactobacillus salivarius*, *Lactobacillus delbrueckii* subsp. *bulgaricus*, *Streptococcus thermophilus*	Antioxidant	[183]
Ginseng fermented milk	*Lactobacillus acidophilus, Bifidobacterium longum* subsp. *longum*, *Streptococcus thermophilus*	Antibacterial effect	[184]

“-” indicates absence.

## 6. Conclusions and Future Perspectives

**Figure 4:**
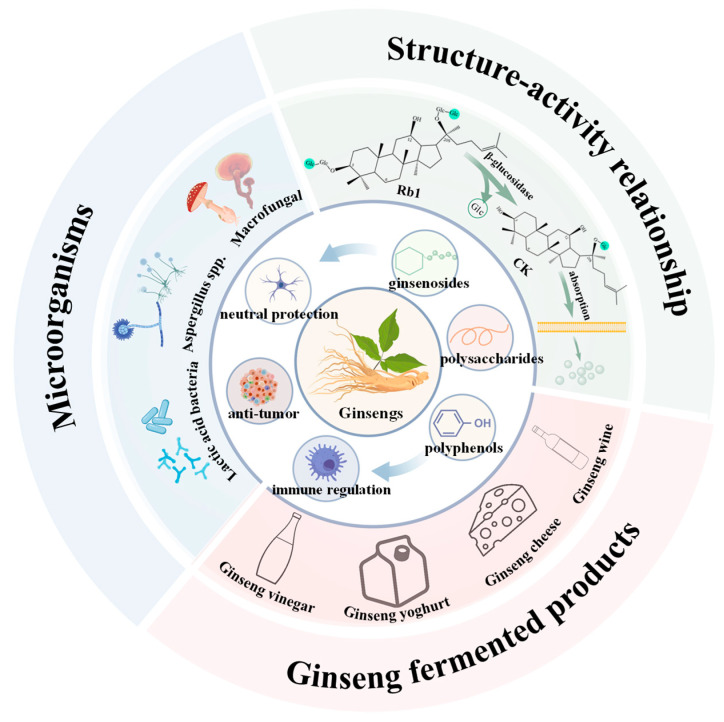
The impact of microbial fermentation on the active ingredients of ginseng and its fermented products. Figure was created with BioGDP.com [185].

As illustrated in Figure 4, this paper provides a systematic summary of the characteristics of lactic acid bacteria, *Aspergillus* spp., and macrofungi involved in ginseng fermentation, integrating findings from bibliometric analysis with experimental research progress. It examines the impact of fermentation on the structure–activity relationship of key active ginseng components, including ginsenosides, ginseng polysaccharides, and polyphenols. Additionally, the paper summarizes and generalizes fermented ginseng products, including ginseng wine, fermented milk, and vinegar.

However, current research continues to face numerous challenges. Firstly, there is a lack of systematic framework for the screening of microbial strains and metabolic regulation. The transformation efficiency of different strains when utilizing the same substrate exhibits considerable variation, and the mechanisms underlying the generation of intermediate products remain poorly understood. Secondly, existing studies primarily focus on individual active ingredients, such as ginsenosides, with limited exploration of the synergistic effects and structure–activity relationship among multiple components, like polysaccharides and polyphenols. This limitation hinders the development of synergistic products. Additionally, the standardization of fermentation processes remains low; there is a lack of established norms governing strain growth conditions, substrate pretreatment, and product stability control. These factors collectively hinder industrial-scale production.

Against the backdrop of rapidly advancing AI, AI holds significant potential for accelerating future research. These models can expedite the breeding of highly efficient transformation strains (such as high-yield β-glucosidase bacteria), elucidate the pathway for intermediate products, and reduce the research and development cycle by orders of magnitude. Furthermore, by integrating synthetic biology and machine learning, AI can facilitate analysis of the structure–activity relationship and the synergistic effects of multiple components, like polysaccharides and polyphenols, thereby advancing the development of functional products.

## Figures and Tables

**Table 1:** Keyword occurrence frequency statistics.

Classification	Keyword	Frequency
Ginseng	*Panax ginseng*	270
	Red ginseng	62
	Korean red ginseng	28
	Fermented red ginseng	27
	*Panax notoginseng*	64
	Notoginseng	6
	American ginseng	23
	*Panax quinquefolius*	8
	Black ginseng	5
	Fermented black ginseng	5
Microorganism	Lactic acid bacteria	20
	*Lactobacillus*	4
	*Lactiplantibacillus plantarum*	13
	*Limosilactobacillus fermentum*	3
	*Aspergillus niger*	16
	*Aspergillus tubingensis*	5
	*Ganoderma lucidum*	10
	*Saccharomyces cerevisiae*	7
	*Bacillus amyloliquefaciens*	3
	*Bacillus subtilis*	3
Active ingredient	Compound k	181
	Ginsenoside compound k	11
	C K	7
	Ginsenosides	105
	Rb1	134
	Ginsenoside Rb1	26
	Rd	31
	Ginsenoside Rd	17
	Rg3	30
	Ginsenoside Rg3	2
	Rh2	9
	20(s) ginsenoside Rh2	2
	F2	11
	Ginsenoside Rb2	2
	Rc	5
	*Panax notoginseng* saponins	11
	Polysaccharides	19
	Phenolic compounds	12

**Table 3:** Transformation pathways of Rb1.

NO.	Transformation Pathway
1	Rb1→Gyp17→Gyp75→C-K→PPD
2	Rb1→Gyp17→F2→C-K→PPD
3	Rb1→Gyp17→F2→Rh2→PPD
4	Rb1→Rd→F2→C-K→PPD
5	Rb1→Rd→F2→Rh2→PPD
6	Rb1→Rd→Rg3→Rh2→PPD

**Table 4:** Transformation pathways of ginsenoside Re.

NO.	Transformation Pathway
1	Re→Rg1→Rh1→PPT
2	Re→Rg1→F1→PPT
3	Re→Rg2→Rh1→PPT

**Table 5:** Transformation pathways of different types of ginsenosides.

Microorganisms	Substrates	Conversion Rates	Transformation Pathways	References
*Aspergillus niger* XD101	Rb1	94.4%	Rb1→Rd→F2→C-K	[110]
*Endophytic bacterium* G9y	Rc	98%	Rc→Rd	[127]
*Pestalotiopsis biciliata*	Rb1	-	Rb1→Rd→F2→C-K	[128]
*Cordyceps militaris* C03	Rg1	54.9%	Rg1→Rh1	[121]
			Rg1→F1	
	Rc	83.44%	Rc→Rd→Rg3→CK	
			Rc→CMc	
*Lentilactobacillus buchneri* URN103L	Rb1	-	Rb1→Rd→Rg3	[111]
*Lactiplantibacillus plantarum* S165	R1	82.85%	R1→20(S/R)-R2	[129]
*Dekkera anomala* YAE-1	Rb1	-	Rb1→Rd	[130]
*Aspergillus niger* JGL8	Gypenoside	-	Gyp-V→Rd→F2	[131]
			Gyp-XVII→F2	
*Penicillium decumbens*	Rb1	-	Rb1→Gyp17→F2→C-K	[29]
			Rb1→Rd→F2→C-K	
			Rb1→Rd→Rg3→Rh2	
*Flavobacterium* sp. GE 32	Rb1	-	Rb1→Gyp-XVII	[132]
			Rb1→Rd→Rg3	
*Microbacterium trichothecenolyticum* KCTC 19343	Rb1	-	Rb1→Rd→Rh2	[30]
*Endophytic fungi* GE 17-18	Rb1	-	Rb1→Rd→F2→C-K	[133]
*Microbacterium* sp. GT35	Re	72%	Re→Rg2	[134]
	Rg1	-	Rg1→Rh1	
*Absidia coerulea* AS 3.2462	Rg1	-	Rg1→F1	[135]
*Cordyceps Sinensis* CICC14017	Rg1	82.5%	Rg1→20(S/R)-Rh1→25-OH-20(S/R)-Rh1	[122]
*Cellulosimicrobium cellulans* sp. 21	Rb1	-	Rb1→Rd→Rg3→Rh2→PPD	[136]

“-” indicates absence.

## Data Availability

No new data were created or analyzed in this study. Data sharing is not applicable to this article.
